# Training and business performance: the mediating role of absorptive capacities

**DOI:** 10.1186/s40064-016-3752-6

**Published:** 2016-12-05

**Authors:** Felipe Hernández-Perlines, Juan Moreno-García, Benito Yáñez-Araque

**Affiliations:** 1 Department of Business Administration, University of Castilla-La Mancha, Toledo, Spain; 2 Department of Information Systems and Technologies, University of Castilla-La Mancha, Toledo, Spain

**Keywords:** Training, Business performance, Dynamic capabilities, Absorptive capacity, Resource-based view, Mediating effect, PLS-SEM, Fs/QCA

## Abstract

**Background:**

Training has been the focus of considerable conceptual and empirical attention but is considered a relevant factor for competitive edge in companies because it has a positive impact on business performance. This study is justified by the need for deeper analysis of the process involving the transfer of training into performance. This paper’s originality lies in the implementation of the absorptive capacities approach as an appropriate conceptual framework for designing a model that reflects the connection between training and business performance through absorptive capacities.

**Results:**

Based on the above conceptual framework and using the dual methodological implementation, a new method of analyzing the relationship between training and performance was obtained: efforts in training will not lead to performance without the mediation of absorptive.

**Conclusions:**

Training turns into performance if absorptive capacities are involved in this process. The suggested model becomes an appropriate framework for explaining the process of transformation of training into organizational performance, in which absorptive capacities play a key role. The findings obtained can go further owing to fs/QCA: of the different absorptive capacities, that of exploitation is a necessary condition to achieve better organizational performance. Therefore, training based on absorptive capacity will guide and facilitate the design of appropriate human resource strategies so that training results in improved performance. This conclusion is relevant for the development of a new facet of absorptive capacities by relating it to training and resulting in first-level implications for human resource management.

## Background

Training is considered a relevant factor for competitive edge in companies because it has a positive impact on business performance (Alavi and Leidner 2001; Barba-Aragon and Sanz-Valle 2013; Bassi et al. 2002; Bartel 2000; Black and Lynch 1996; Bryan 2006; Hansson 2007). Although training has been the focus of considerable conceptual and empirical attention in the business literature, the process of transforming training into improved business performance remains unclear.

Given the lack of similar studies, this paper seeks to illuminate how training is transformed into improved performance. In this regard, in line with the argument proposed by Hernández-Perlines and Yáñez-Araque (2015), the main objective is the implementation of the dynamic capabilities approach as an appropriate conceptual framework for determining the connection between training and business performance through absorptive capacities. From the above conceptual framework and with the implementation of a dual research methodology, partial least squares-structural equation modeling (PLS-SEM) and fuzzy set qualitative comparative analysis (fs/QCA), a significant contribution is achieved: efforts in training will not lead to improved performance without the mediating role of absorptive capacities, with operating capacity as the condition required for this mediating effect.

The literature has extensively analyzed the impact of training on organizational performance: training with performance, training with competitive advantage, training with business requirements, etc. (Hernández-Perlines and Yáñez-Araque 2015). However, some authors argue that, despite the obvious significance of training, which is difficult to refute from any perspective, it does not have the same impact on science or on business practice. In part, the reason is the lack of generally accepted explanatory models and the inability to make rigorous predictions in this regard (Araujo and Guisan 2006). Some authors suggest that absorptive capacity (ACAP) helps explain how the process of transformation of training into organizational performance occurs (Spence 1973, Taubman and Wales 1973) because it is a mediating variable.

The analysis of both training and ACAP can be performed based on resource-based theory (Barney 1991; Grant 1996; Sveiby 1997; Wernerfelt 1984), knowledge management (Kogut and Zander 1992; Nonaka and Takeuchi 1995), intellectual capital (Bueno et al. 2004; Bontis 2001) and the dynamic capabilities approach (Easterby-Smith and Prieto 2008; Teece et al. 1997). This paper aims to integrate them to adequately conceptualize both training and ACAP.

Most studies that analyze the impact of training on business performance (Wright et al. 1994a, b; Kamoche 1996; Mueller 1996; Barney and Wright 1998; Bassi et al. 1998; Lee and Yang 2000; Hitt et al. 2001) are based on the role that it plays in the development of human capital (Becker 1964; Fahy 2000) and in organizational knowledge (Alavi and Leidner 2001; Bollinger and Smith 2001).

As a result, companies may believe that the mere establishment of training may be enough to improve performance, but this is not true because it is not possible to make rigorous predictions in this regard (Araujo and Guisan 2006). In this sense, ACAP is an element that strengthens the relationship between training and business outcomes.

Zahra and George (2002) reconceptualize ACAP as a multidimensional construct that involves the dynamic capability of creating and using knowledge that is related to the company’s capability of being competitive. According to these authors, ACAP has four dimensions that are grouped into two types of capacities: potential ACAP (acquisition and assimilation) and performed ACAP (transformation and exploitation).

In the early days of the ACAP concept, the first connection between training and ACAP could already be found. Cohen and Levinthal (1990) stated that “the ACAP concept is better developed by examining the cognitive structures underlying learning”(p. 129) Hernández-Perlines and Yáñez-Araque (2015) undertake a theoretical journey through the chain relationships of different complementary concepts that help understand how training has an impact on performance through the ACAP process, further reinforcing the interconnection between training and ACAP.

The literature provides studies that analyze the moderating effect of ACAP: Engelen et al. (2014), between entrepreneurial orientation and the environment; Ahlin et al. (2014), between social networks and innovation; and external knowledge flows (Escribano et al. 2009). On the other hand, other studies analyze the mediating impact of ACAP; these include studies by Aljanabi et al. (2014), between factors of organizational support and technological innovation; Leal-Rodríguez et al. (2014), on innovation performance; Saenz et al. (2014), on buyer–seller relationships; Adisa and Rose (2013), on knowledge transfer; Liu et al. (2013), on information technology capabilities on business outcomes; Zhou and Wu (2010), on technology management capability and new product development services; Chen and Huang (2009), on human resource practices and the results of organizational innovation; and Van den Bosch et al. (1999), on new knowledge and organizational adaptation. All this leads to the primary hypothesis of this work:

### **H**_**1**_

ACAP positively mediates the relationship between human resource training and business performance.

Mathieu and Taylor (2006) define mediation as a variable that behaves as a mediator (in our case, ACAP), enhancing or reducing the influence of a precedent variable (in our case, training) on a criterion variable (in our case, organizational performance). Therefore, the mediating effect helps divide the primary hypothesis previously raised into two sub-hypotheses (fuzzy propositions to operationalize them when implementing fs/QCA).

The first is linked to training with ACAP. It can be stated that the acquisition of new external knowledge precedes ACAP (Van den Bosch et al. 1999; Zahra and George 2002), with training being the input of ACAP (Cohen and Levinthal 1990; Yahya and Goh 2002). On this basis, the following research hypothesis is proposed:

### **H**_**2**_

Training has a positive impact on ACAP.

Several studies have focused on business performance as a result of ACAP. Indeed, the empirical results show a significant positive relationship between ACAP and business performance (Bergh and Lim 2008; Jansen et al. 2005; Lane et al. 2006; Mowery et al. 1996; Mukherjee et al. 2000; Todorova and Durisin 2007; Tsai 2001; Yeoh 2009; Zahra and George 2002). Accordingly, we propose the following research hypothesis:

### **H**_**3**_

ACAP has a positive impact on performance.

## Methods

This study analyzes the process involving the transfer of training in performance, through consideration of the absorptive capabilities. This paper’s originality lies in the implementation of the absorptive capabilities approach as an appropriate conceptual framework for designing a model that reflects the connection between training and business performance through absorptive capacities.

**Fig. 1:**
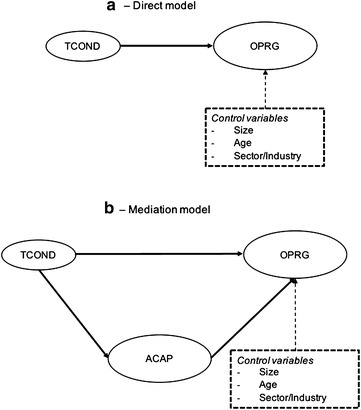
Structural model

The proposed model is shown in Fig. 1. This is a structural model, which can be broken down into two: direct model and mediation model.

### Sample

For the sample selection, the database of Spanish companies compiled by SABI (Iberian Balances Analysis System database) was used. This database provides financial and business information of more than 1.3 million Spanish and Portuguese companies. Before the creation of the final questionnaire, a pretest was performed with a group of six companies. The final questionnaire, called STraDyCaF © (www.stradycaf.org), is a self-administered questionnaire with Likert scale questions (1–7) and other open-ended questions that are more specific to the semi-structured interview. The survey was distributed and administered between May and December 2014 through a web-based tool, LimeSurvey version 2.05+. Through this open source web application, designed for developing and managing surveys and populations, the survey and an institutional letter of introduction presenting it were sent to each participant (i.e., CEO, human resources director, head of training) as an email with a personalized link. To encourage participation and improve the response rate, a simple design was provided allowing for responses from mobile devices, in addition to a text to speech assistant, similar to CATI (computer assisted telephone interviewing) systems. A representative sample of Spanish companies was selected on the basis of simple random sampling, including those with 50 workers and not confined to any particular sector. At the end of the process, as shown in Table 1, 112 valid cases were used to analyze the data in PLS-SEM. Regarding the other analysis method, 25 cases were randomly selected within the valid cases.

**Table 1:** Data sheet of the PLS-SEM field and fs/QCA fieldwork

Target population (universe)	Spanish companies with 50 or more workers belonging to any sector except public administration, the agricultural sector, fishing, activities of households and extraterritorial bodies^a^
Geographical scope	The entire national territory/Spanish national territory
Analysis unit/sampling unit	The company
Population census^b^	22,013
Sample size/response rate (PLS-SEM)	112 valid surveys/7.18%
Sampling procedure	Simple random sampling without replacement
Confidence level	95%; z = 1.96; p = q = 0.50; α = 0.05
Sampling error	9.24%
Key informant	CEO, head of training and/or human resources director
Date of fieldwork/data collection	Between May and December 2014
Sample size (fs/QCA)	Random group of 25 cases from the data set

^a^The excluded economic sectors/activities correspond to the national classification of economic activities (CNAE) 2009 and Statistical Classification of Economic Activities in the European Community (NACE) Rev. 2 sections: (A) agriculture, livestock breeding, forestry and fishing; (O) public administration and defense, compulsory social security; (T) activities of households, such as employers of domestic staff, activities of households, such as producers of goods and services for one’s own use; and (U) activities of extraterritorial organizations and bodies

^b^ *Source*: DIRCE 2014 (Central Companies Directory, Jan 1, 2014)

### Measuring variables

To undertake the study and achieve its purpose, the following three variables were considered: training, absorptive capacities and organizational performance. These variables were measured as described below:

#### Training

Training was measured using the validated five-item scale suggested by Castañeda and Fernández (2007), and a Likert scale (1–7) was adopted. In this study, training is considered a first-rate A-type composite for data analysis in PLS.

#### Organizational performance

In this study, organizational performance is considered a dependent variable. A Likert scale (1–7), which was validated by Camisón and Villar-López (2010), was used for its measurement. This scale considers two dimensions of organizational performance: first, economic performance (five items) and, second, performance in satisfaction (four items). This variable was considered a second-order A-type composite in PLS.

#### Absorptive capacity

The study considers ACAP as a multidimensional variable, in line the suggestions by Cohen and Levinthal (1990) and Lane et al. (2006), by using the four-dimensional scale validated by Flatten et al. (2011), which assesses the extent to which a company is engaged in activities of knowledge acquisition (acquisition, three items), assimilates the acquired information with the existing knowledge (assimilation, four items), transforms the newly adapted knowledge (transformation, four items) and commercially exploits the transformed knowledge to its competitive advantage (exploitation, three items). For all items, a Likert scale (1–7) was used. For the PLS method, absorptive capacity was operationalized as a second-order A-type composite.

#### Control variables

The control variables used in this study were categorical. The main sector of activity, the size measured by the number of workers and the age of the company, i.e., the number of years since its establishment, were considered.

### Combination of methods for data analysis

One of the most significant aspects of this work is the combination of different research methodologies (qualitative and quantitative). In addition to its complementarity, this combination gives validity and robustness to the research conducted (Goertz and Mahoney 2013).

In this paper, we propose two methods. First, the PLS-SEM method is suggested. Nowadays, PLS is an established technique of structural equation analysis, which has been used in a variety of investigations into organization and strategic management (Robins et al. 2002; Tsang 2002). The PLS technique takes a component-based model for estimation of measurement and structural model approach. Its main purpose is the prediction of the dependent variables, and to quantify the direct and indirect effects of variables on each other. This technique is less demanding on the minimum requirements about the sample size, the nature of the measurement scales and the distribution of the observables variables, compared to the tools of structural equation models based on the covariance as LISREL or EQS (Haenlein and Kaplan 2004). PLS path analysis allows to evaluate mediation models and tests mediation hypotheses, using the bootstrapping method (Hayes et al. 2011). As a result, the study uses the bootstrapping method to test mediation, i.e. the importance of indirect effects. Bootstrapping is a nonparametric re-sampling procedure for the analysis of both simple and multiple mediation. It imposes no assumptions about the distribution of variables or the distribution of the sample and can be applied to small sample sizes with more confidence. Therefore, this approach is perfectly suited for the PLS-SEM method (Hair et al. 2014). Authors like MacKinnon et al. (2004); Hayes and Scharkow (2013) have proven that this method leads to higher performance and higher levels of statistical power than traditional ways of testing mediation hypotheses, such as the Sobel test (1986), which requires a normal distribution of indirect effects in the sample along with other problems that prevent its application with PLS. The PLS-SEM method is particularly interesting in the early stages of theory development (Ringle et al. 2012; Gefen et al. 2011), when researchers include scales that were tested and validated in previous studies or a relatively small sample size (Reinartz et al. 2009), or, finally, when models are very complex (Hair et al. 2014). The PLS-SEM method is not without limitations (e.g., symmetrical causal relationships, net effects, etc.), most frequently due to issues of multiple regression analysis (MRA) and structural equation models (SEMs) (Woodside 2013; Skarmeas et al. 2014). The statistical program SmartPLS 3.2.5 was used to apply PLS-SEM (Ringle et al. 2015).

We propose using fs/QCA, which is suggested to overcome the above constraints. This technique is a useful qualitative method for the study of social phenomena with a small number of data, in which uncertainty can be properly managed (Ragin 2000, 2008). The fs/QCA 2.5 software is used to apply fs/QCA (Ragin and Sean 2014).

## Results

The results were structured according to the implementation of the various methodologies suggested.

### Results for PLS-SEM

Two distinct stages were developed to interpret and analyze the suggested model in PLS-SEM (Barclay et al. 1995): (1) analysis of the measurement model; and (2) analysis of the structural model. This sequence ensures that the suggested measuring scales are valid and reliable.

#### Analysis of the measurement model

Table 2 shows the parameters associated with the evaluation of the measurement model. All factor loading values are greater than 0.5, which is considered acceptable by Barclay et al. (1995) and Chin (1998). Additionally, the values of composite reliability and of the average variance extracted (AVE) exceed the recommended limits of 0.7 and 0.5, respectively (Nunnally 1978; Fornell and Larcker 1981). The values obtained support the convergent validity of the A-type composites considered. Finally, to ensure discriminant validity, the correlations between each pair of composites did not exceed the value of the square root value of the AVE of each composite. In addition, following the HTMT criterion (Henseler et al. 2015) all variables achieve discriminant validity (Table 3).

**Table 2:** Correlation matrix, reliability estimates, convergent and discriminant validity and descriptive statistics

Composite/measures	AVE	Composite reliability	1. ACAP	2. TCOND	3. ORGP
1. Absorptive capacity (ACAP)	0.66	0.83	0.81	0	0
2. Training conditions (TCOND)	0.67	0.91	0.77	0.82	0
3. Organizational performance (ORGP)	0.81	0.9	0.73	0.53	0.90
Cronbach’s alpha	–	–	0.75	0.87	0.78
Mean	–	–	5.04	5.32	4.90
Typical deviation	–	–	0.91	1.18	1.01

The means were evaluated based on the average scores of the different first-order composites that comprise each of the second-order composites considered. The correlations are those of the second-order composites according to the CFA outputs. The elements in the diagonal are the square root of the AVE

**Table 3:** Heterotrait-monotrait ratio (HTMT)

	1. ACAP	2. TCOND	3. ORGP
1. Absorptive capacity (ACAP)			
2. Training Conditions (TCOND)	0.634		
3. Organizational Performance (ORGP)	0.623	0.62	

#### Analysis of the structural model

Once the convergent and discriminant validity of the measurement model was secured, the relationships between different variables were tested. To determine the different effects, the steps suggested by Hair et al. (2014) were followed to apply the approach by Preacher and Hayes (2004) in the mediation model.

In order to evaluate the statistical significance of path coefficients, standard errors and t-statistics (pseudo-parametric test) are generated from the bootstrapping procedure (5000 subsamples) (Hair et al. 2011). Similarly, the nonparametric approach (not based on any distribution) reports bootstrap confidence intervals of beta weights and indirect effects for mediation hypotheses. According to Henseler et al. (2009): if the confidence interval estimated for the path coefficient does not contain zero, it means that the estimated path coefficient is significantly different from zero, where the percentage (confidence level) is used to calculate confidence intervals. Likewise, the significance of confidence intervals for mediation hypotheses is interpreted, as discussed below. In particular, the percentile approach is applied to the bootstrap re-sampling with a 95% confidence. The advantage of this approach is that it does not presuppose any distribution of data (Chin 2010).

**Fig. 2:**
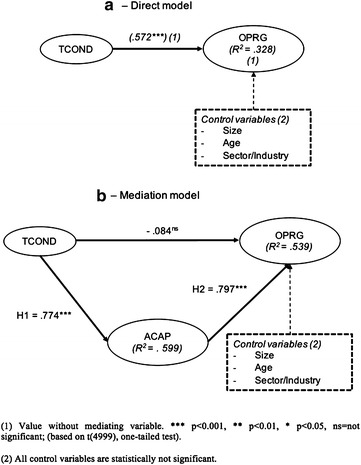
Results of structural model

First, the direct effect between training (TCOND) and organizational performance (ORGP) is observed (model A). To that end, the bootstrap method (5000 sub-samples) is applied. This effect is positive and significant (β = 0.572; *t* value surpasses the minimum level indicated by Student’s t-distribution with one tail and n-1 degrees of freedom, where n is the number of subsamples with a 99.9% confidence level. Therefore, the probability of being wrong in rejecting the hypothesis is null: p < 0.001. This result is reinforced by applying the percentile method on bootstrap re-sampling in a 95% confidence interval (Table 4a; Fig. 2a). The second step includes the effect of the mediating variable (ACAP) (model B). The indirect effect is positive and significant (between TCOND and ACAP H_2_: β = 0.774, p < 0.001; and between ACAP and ORGP H_3_: β = 0.797; p < 0.001; confidence intervals does not include zero), with a suppressor effect, which characterizes the sign change of the direct relationship after the mediator variable have been included (Hair et al. 2014) (β = −0.084; p > 0.05; confidence interval includes zero; not significant) (Table 4b). The explained variance (VAF) is greater than 1 (specifically, 1.16); thus, the direct effect is eliminated. In the model, the positive and significant relationship without the mediating variable becomes negative and not significant after the inclusion of the mediating variable (β = −0.084; p < 0.001). Therefore, there is full mediation (Baron and Kenny 1986): absorptive capacity fully mediates the relationship between training and organizational performance (hypothesis H1, Fig. 2). In addition, the evaluation of the structural model shows a good fit (GoF: 0.62), high consistency (R_2_: 0.54 for model b is still better than model a, which is additional support for the mediating role of absorptive capacity), accuracy predictive relevance (Q_2_: ACAP 0.31; Q_2_: ORGP: 0.44): all parameters progressively improve from model A to B: model B is better than Model A. Following Henseler et al. (2014), the standardized root mean square residual (SRMR) for a composite factor model is determined. This provides the exact fit of the composite factor model, thus constituting a confirmatory composite analysis (Felipe et al. 2016). Model A (total effect) achieves a SRMR composite factor model of 0.073, which means an appropriate fit assuming the usual cut-off of 0.08 (Hu and Bentler 1999). However, the SRMR composite factor model for Model B is still better: 0.068. This would imply an additional support for the mediating role of absorptive capacity (Table 5). The analysis of the control variables does not reveal any significant path; therefore, they are excluded from the model.

**Table 4:** Causal relationships: total, direct and indirect effects

Direct effects	Path coefficient (β)	Point estimate	t-value (bootstrap)	Percentile 95% confidence intervals		Support
				Lower	Upper	
*Total and indirect effects/mediation hypotheses*						
(a) Model A (SRMR cfm = 0.073)						
Total effect of Training on O. Performance (*c*) (Model A: model with total effect)	0.5723***		8.3753	0.4439	0.7151	Yes
(b) Model B (SRMR cfm = 0.068)						
Training → O. Performance = *c’* (direct effect of Training on O. Performance)	−0.0836^ns^		0.6174	−0.3278	0.2025	Not
H1 = Training → ACAP → O. Performance = *a*_1_ *b*_1_ (via ACAP) (total indirect effect of Training on O. Performance)		0.6164		0.4659	0.7522	Yes
H2 = Training → ACAP = *a*_1_	0.7737***		21.4803	0.7003	0.8416	Yes
H3 = ACAP → O. Performance = *b*_1_	0.7967***		7.8783	0.5721	0.9753	Yes

*ACAP* absorptive capacity, *O. Performance* organizational performance

t(0.05, 4999) = 1.645158499, t(0.01, 4999) = 2.327094067, t(0.001, 4999) = 3.091863446

*** *p* < 0.001, *ns* not significant (based on t (4999), one-tailed test)

**Table 5:** Summary of evaluation of the structural models

Comparison of models effects on endogenous variables	Direct effects^a^	Explained variance	R^2^ value	Q^2^ value	ΔR^2^	f^2^	GoF
Model A							0.4900
Training → O. Performance = *c* (only direct effect)	Sig.		0.3275	0.1768			
Model B							0.6241
Endogenous latent variables							
ACAP			0.5987	0.3147			
H2 = Training → ACAP = *a*_1_	Sig.	0.5987					
O. Performance (mediated by ACAP)			0.5387	0.4417	0.2112	0.4578	
Training → O. Performance = *c’*	Nsig.	0.0445^b^					
H3 = ACAP → O. Performance = *b*_1_	Sig.	0.5832					

^a^Sig. denotes a significant direct effect; Nsig. denotes a nonsignificant direct effect

^b^This sum (0.0446 + 0.5832) is not equal to R^2^ (0.5387), note that the contribution of training to the explained variance of O. Performance is negative, but very small. This commonly occurs when the sign of the zero-order correlation is the opposite of the sign of the path coefficient (Menard 2009). In Model B, the path coefficient *c’* is negative, but very small and nonsignificant direct effect

#### Predictive validity of the PLS model using holdout samples

Assessment of the model’s predictive validity using retention samples completes the PLS-SEM analysis in this study (Becker et al. 2013; Cepeda et al. 2016; Evermann and Tate 2016; Hernández-Perlines 2016). Randomly assigning numbers to cases divides the original sample into two subsamples: the training sample (two-thirds of the total population: 75 cases), and the holdout sample (one-third of the total population: 37 cases). The following steps are the estimation of parameters in the training sample and the standardization of data for each composite in the holdout sample, bearing in mind that absorptive capacity and organizational performance are second-order composites. This step allows the creation of each indicator from a linear combination of its respective composites in the holdout sample, using the weightings from the training sample. In the study applies the new linear combinations to the first-order dimensions and the second-order composites. The second-order composites and the composite training are therefore as follows, with weights obtained from training sample:

$$\begin{aligned} {\text{ACAP}} & = 0.232{\text{ AC}}_{\text{std}} + \, 0.268{\text{ AS}}_{\text{std}} + \, 0.303{\text{ TRANSF}}_{\text{std}} + \, 0.451{\text{ EX}}_{\text{std}} \\ {\text{ORGP}} & = 0.505{\text{ ORGPEC}}_{\text{std}} + 0.647{\text{ ORGPS}}_{\text{std}} \\ \end{aligned}$$

$$\begin{aligned} {\text{TCOND}} & = 0.279\;{\text{TCONDFQ1}}_{\text{std}} + \, 0.236{\text{ TCONDFQ2}}_{\text{std}} + \, 0.261\;{\text{TCONDFQ3}}_{\text{std}} \\ & \quad + \, 0.13\;{\text{TCONDFQ4}}_{\text{std}} + \, 0.271\;{\text{TCONDFQ5}}_{\text{std}} \\ \end{aligned}$$

The next step consists of standardizing the values of each composite in the holdout sample. The path coefficients of the training sample then serve to complete score prediction for each composite in the holdout sample, thus yielding the following expression:

$${\text{ORGP}}_{\text{pred}} = {-}0.043\,{\text{TCOND}}_{\text{std}} + \, 0.811\,{\text{ACAP}}_{\text{std}}$$

For the composite ORGP, R^2^ is a square correlation of the predicted ORGP and the ORGP in the standardized model.

$${\text{R}}^{2} = \left( {{\text{corr }}\left( {{\text{ORGP}}_{\text{pred}} ,{\text{ ORGP}}_{\text{std}} } \right)} \right)^{2} = \, 0.634$$

Finally, to complete the assessment of the model’s predictive validity, the study compares the R^2^ for the holdout sample with the R^2^ for the training sample (to two decimal places).

$${\text{R}}_{\text{holdoutsample}}^{2} = \, 0.63$$

$${\text{R}}_{\text{Trainingsample}}^{2} = \, 0.60$$

The two values are close, thus indicating that the model has predictive validity.

### Results for fs/QCA

Regarding the implementation of this method, first, we calibrate the data. To do so, we use the Calibrate function (var, full, mid, non-full) with values of 7.5 and 3, respectively. Regarding the input condition (exp), the function *Expls* = *Calibrate (exp, 7, 5, 3)* is used, with fs being the fuzzy condition (Zadeh 1965; Moreno-Garcia et al. 2014) that is added to the input condition.

The study results applied to hypothesis H2 show that, in all cases, consistency exceeds the value of 0.65, i.e., the “usually necessary conditions,” becoming “almost always necessary conditions” in the case of the following conditions: *condfq2fs*, *condfq3fs*, *condfq4fs* and *condfq5fs*, although none of them is individually sufficient. On the other hand, all negated conditions are less than 0.50, except ~ *condfq1fs*, which has a value of 0.55 (“more often than unnecessary conditions”) (Table 6).

**Table 6:** Analysis of the necessary conditions for H_2_ and H_3_

H_2_: Analysis of necessary condition H_2_ Outcome: ACAP			H_3_: Analysis of necessary condition H_3_ Outcome: ORGP		
	Consistency	Coverage		Consistency	Coverage
condfq1fs	0.771714	0.783151	adqfs	0.779099	0.715289
condfq2fs	0.853958	0.811541	asqfs	0.778250	0.728140
condfq3fs	0.965411	0.733645	transffs	0.790994	0.799142
condfq4fs	0.811683	0.764110	exqfs	0.951572	0.700876
condfq5fs	0.906995	0.685648	~adqfs	0.592183	0.572250
~condfq1fs	0.551883	0.589491	~asqfs	0.613424	0.581320
~condfq2fs	0.486549	0.559682	~transffs	0.586236	0.516854
~condfq3fs	0.371253	0.612944	~exqfs	0.430756	0.562084
~condfq4fs	0.492698	0.573345			
~condfq5fs	0.291314	0.486521			

**Fig. 3:**
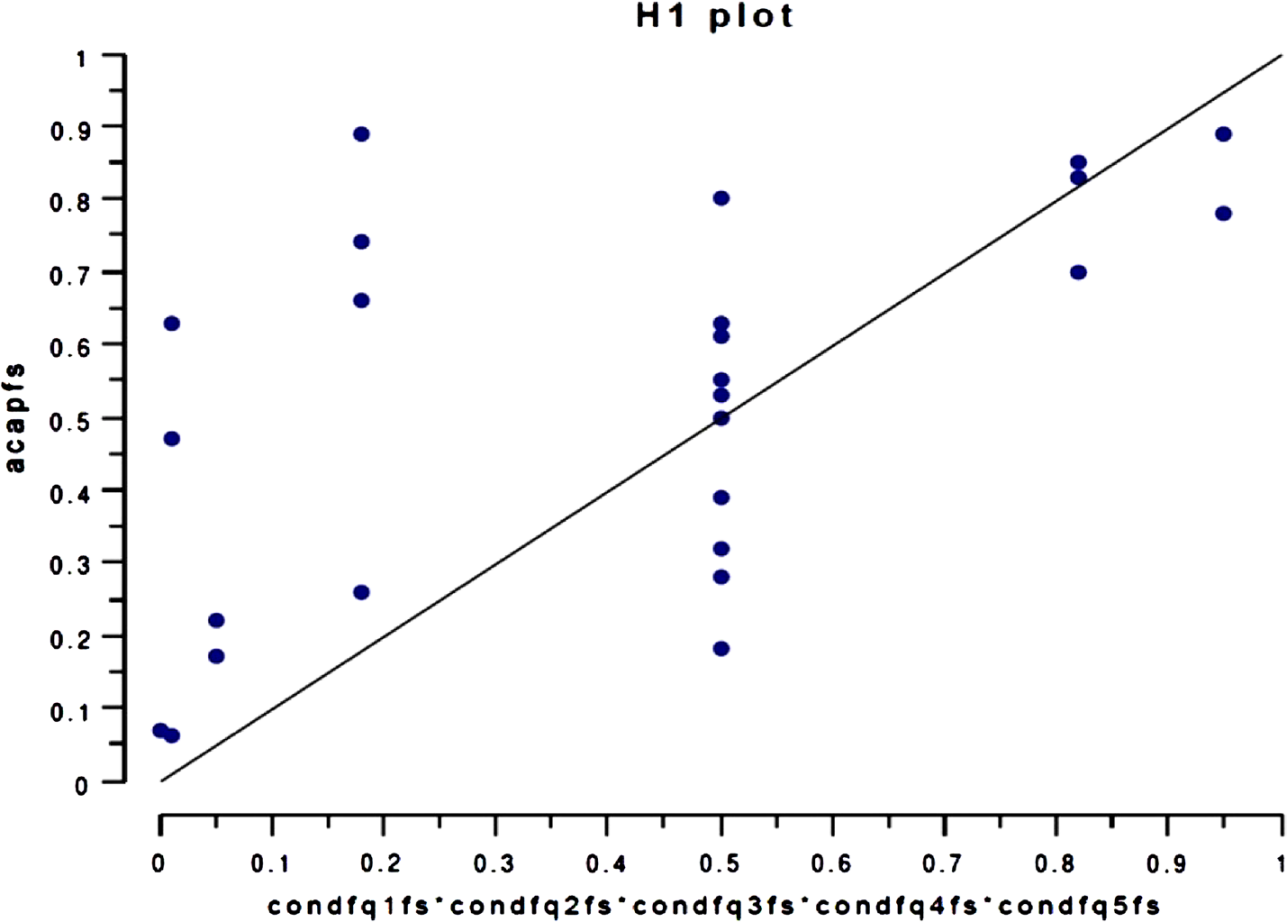
Graphic representation of condfq1fs*condfq2fs*condfq3fs*condfq4fs*condfq5fs against acapfs

In addition, there is a combination of conditions that guarantee the level of ACAP (Table 7). This combination uses all input combinations, obtaining a strong consistency (0.88, which is higher than the minimum value of 0.75 recommended by Ragin 2008). On the other hand, the coverage value is sufficient (0.69). Moreover, membership values are located in the upper triangle (17 out of 25) (Fig. 3), reflecting that the combination of causal conditions is sufficient to ensure ACAP.

**Table 7:** Results when using fs/QCA

Causal configuration	Gross coverage	Single coverage	Consistency
Results for H_2_ complete solution (outcome: ACAP)			
condfq1fs*condfq2fs*condfq3fs*condfq4fs*condfq5fs	0.694081	0.694081	0.884427
Coverage solution: 0.694081			
Consistency solution: 0.884427			
Results for H_3_ complete solution (outcome: ORGP)			
~asqfs*exqfs	0.570093	0.097706	0.773933
transffs*exqfs	0.781648	0.309261	0.855019
Coverage solution: 0.879354			
Consistency solution: 0.776444

Regarding hypothesis H3 (Tables 6, 7), the same was performed. In this case, the necessary conditions are “usually necessary conditions” for everyone, whereas *exqfs* has a consistency value of 0.95, which means that these are “almost always necessary conditions.” The negated condition—*exqfs* has a consistency value of 0.43, which is not a necessary condition. Therefore, it is a necessary but not sufficient condition to ensure performance. Of the sufficient conditions, two combinations guarantee the level of organizational performance because they have a consistency value of 0.78 (which is greater than the value of 0.75 recommended by Ragin 2008). The coverage value is large (0.88). In addition, the *expfs* condition is part of both combinations (as assumed in the analysis of the necessary conditions). The consistency of each combination (0.77 and 0.85) is good. Additionally, in this case, most membership values of the two combinations of causal conditions obtained against the output variable are located in the upper triangle, which reflects that both combinations of causal conditions are sufficient to ensure organizational performance.

## Conclusions

This section is structured around the goals outlined in this study. The first major contribution of this paper is of a methodological nature and involves the use of two complementary methodologies in combination: a qualitative methodology and a quantitative methodology. These two methods of data analysis, PLS-SEM and fs/QCA, validate and corroborate the conceptual model proposed.

The most significant conclusion of this study is that training turns into performance if absorptive capacities are involved in this process. The suggested model becomes an appropriate framework for explaining the process of transformation of training into organizational performance, in which absorptive capacities play a full mediation key role.

However the findings obtained can go further owing to fs/QCA: of the different absorptive capacities, that of exploitation is a necessary condition to achieve better organizational performance. Therefore, training based on absorptive capacity will guide and facilitate the design of appropriate human resource strategies so that training results in improved performance.

This paper is relevant for the development of a new facet of absorptive capacities by relating it to training and resulting in first-level implications for human resource management, which, among others, include the following:

- It is necessary to establish training plans that take into account not only the training requirements but also absorptive capacities.
- Absorptive capacity appears as a form of appropriation of the performance of the companies’ effort in training.
- Training favors the development of dynamic capacities (Teece et al. 1997) and, in particular, absorptive capacities (Yahya and Goh 2002). Companies must evaluate the different absorptive capacities (especially that of exploitation) and then decide what resources are allocated to these capacities (Brettel et al. 2011).

## Abbreviations

ACAP: absorptive capacity
ACAP_std_: absorptive capacity standardized
AC_std_: acquisition capacity standardized
AS_std_: assimilation capacity standardized
AVE: average variance extracted
CATI: computer assisted telephone interviewing
CEO: chief executive officer
CNAE: national classification of economic activities
Corr.: correlation
DIRCE: central Companies Directory
EX_std_: exploitation capacity standardized
Exp: input condition
fs/QCA: fuzzy set qualitative comparative analysis
HTMT: heterotrait-monotrait ratio of correlations
MRA: multiple regression analysis
Nsig.: nonsignificant
ORGP: organizational performance
ORGPEC_std_: economic organizational performance
ORGPS_std_: satisfaction organizational performance
PLS-SEM: partial least squares-structural equation modeling
R^2^: coeficiente de Pearson
SABI: iberian balances analysis system database
SEMs: structural equation models
Sig.: significant
SRMR: standardized root mean square residual
TRANSF_std_: transformation capacity standardized
TCOND: training conditions
Var.: variable
